# Mavacamten Reduces LVOT Obstruction Risk, Enabling Transseptal Mitral Valve-in-Valve for Degenerated Bioprosthesis

**DOI:** 10.1016/j.jaccas.2026.107249

**Published:** 2026-03-14

**Authors:** Alexander Hoss, Fabian Voß, Amin Polzin

**Affiliations:** Division of Cardiology, Pulmonology, and Vascular Medicine, Department of Internal Medicine, Medical Faculty and University Hospital, Heinrich-Heine-University, Düsseldorf, Germany

**Keywords:** LVOT obstruction, mavacamten, neo-LVOT, structural heart intervention, transcatheter mitral valve replacement (TMVR)

## Abstract

**Background:**

Transcatheter mitral valve replacement (TMVR) is an alternative to redo surgery for failed mitral bioprostheses, but left ventricular outflow tract (LVOT) obstruction remains a major limitation.

**Case Summary:**

An 86-year-old woman presented with heart failure due to severe prosthetic mitral restenosis. Screening computed tomography (CT) predicted a critically small neo-LVOT, rendering TMVR prohibitive. Given prohibitive surgical risk and no feasible LVOT obstruction-mitigation procedure, off-label mavacamten 2.5 mg daily was initiated. After 4 months, repeat CT showed neo-LVOT enlargement, enabling transseptal valve-in-valve TMVR without complications. Invasive hemodynamics excluded a relevant LVOT gradient; echocardiography confirmed excellent valve function without LVOT obstruction.

**Why Beyond the Guidelines:**

No guideline-endorsed pharmacologic strategy exists to enlarge predicted neo-LVOT before TMVR.

**Discussion:**

Myosin inhibition may offer a noninvasive LVOT obstruction-mitigation bridge when procedural options are unsuitable.

**Take-Home Message:**

In selected patients with prohibitive neo-LVOT, short-term mavacamten may convert CT screening to a feasible TMVR pathway.

## Visual Summary

**Figure:**
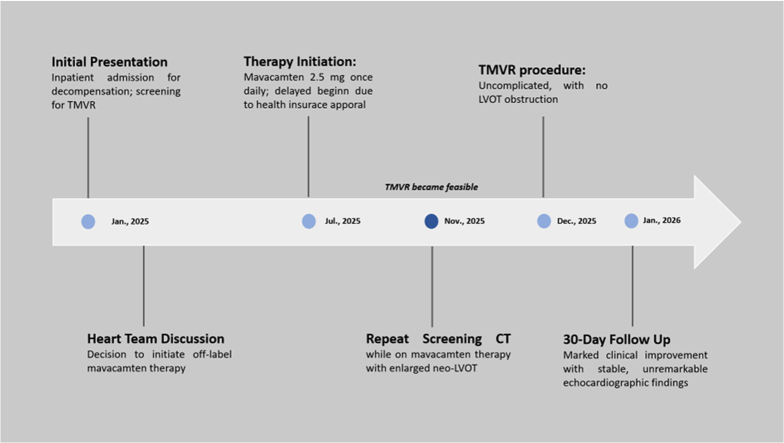

**Visual Summary:**
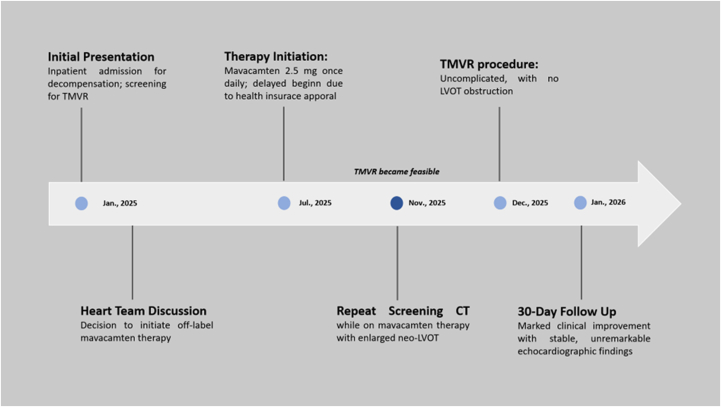
Timeline of the Case CT = computed tomography; LVOT = left ventricular outflow tract; TMVR = transcatheter mitral valve replacement.

Degeneration of surgical bioprosthetic mitral valves is increasingly encountered in contemporary practice. Redo mitral surgery carries substantial morbidity and mortality in elderly, comorbid patients, making transcatheter mitral valve-in-valve replacement (TMViV) an important alternative with favorable outcomes using balloon-expendable valves.^1^ A critical limitation of TMViV is left ventricular outflow tract obstruction (LVOTO), which is associated with high procedural mortality and can be assessed by preprocedural computed tomography (CT) with virtual valve implantation and measuring neo–left ventricular outflow tract (LVOT) area.^2^, ^3^, ^4^, ^5^ Expert consensus emphasizes systematic CT methodology and individualized risk stratification, acknowledging that screening failures due to high risk of LVOTO compromise remain common.^3^^,^^4^

Mavacamten is a selective, oral cardiac myosin inhibitor approved for symptomatic obstructive hypertrophic cardiomyopathy (HCM), requiring structured echocardiographic surveillance given the risk of systolic dysfunction.^6^^,^^7^ Its ability to reduce hypercontractility and induce favorable remodeling in obstructive HCM has already been demonstrated.^8^, ^9^, ^10^

We report off-label mavacamten use to pharmacologically reduce the risk of LVOTO, enabling TMViV in an otherwise ineligible patient.

## Case Summary

An 86-year-old woman with prior cardiac surgery in 2015 (biological mitral valve replacement) for mitral stenosis presented with progressive exertional dyspnea and recurrent decompensations. Relevant comorbidities included atrial fibrillation on chronic anticoagulation, combined post- and precapillary pulmonary hypertension (mean pulmonary arterial pressure: 41 mm Hg, pulmonary arterial wedge pressure: 21 mm Hg, pulmonary vascular resistance: 361 dyn⋅s/cm^5^), and prior concomitant coronary artery bypass grafting performed during the index mitral valve surgery in 2015.

**Figure 1:**
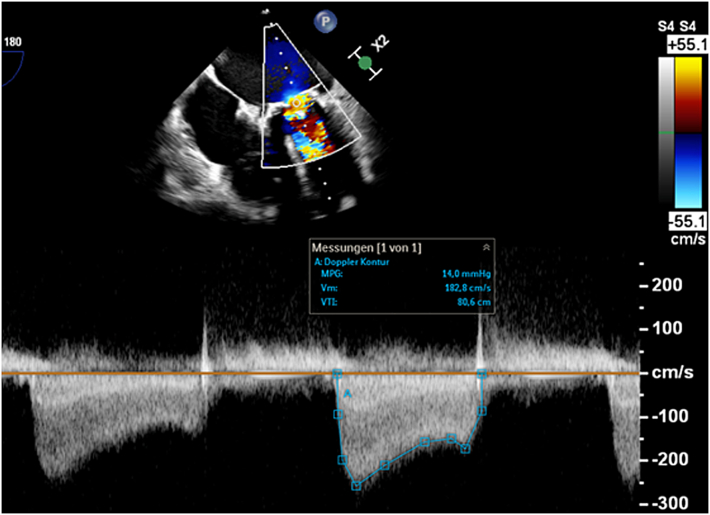
Preintervention Transesophageal Echocardiography Transesophageal echocardiography demonstrating severe stenosis of the bioprosthetic mitral valve; continuous-wave Doppler shows a mean transmitral pressure gradient of 14 mm Hg.

**Figure 2:**
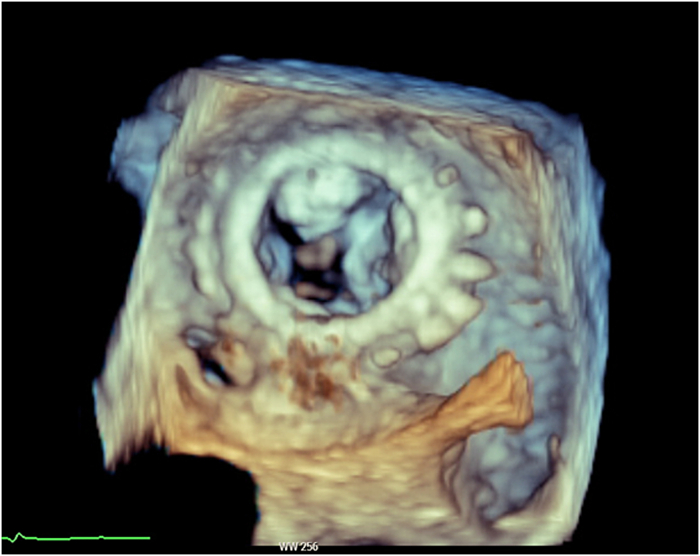
Three-Dimensional View of the Stenotic Mitral Valve Prosthesis Three-dimensional transesophageal echocardiography of severely degenerated bioprosthetic mitral valve showing thickened, sclerotic leaflets with restricted opening.

**Figure 3:**
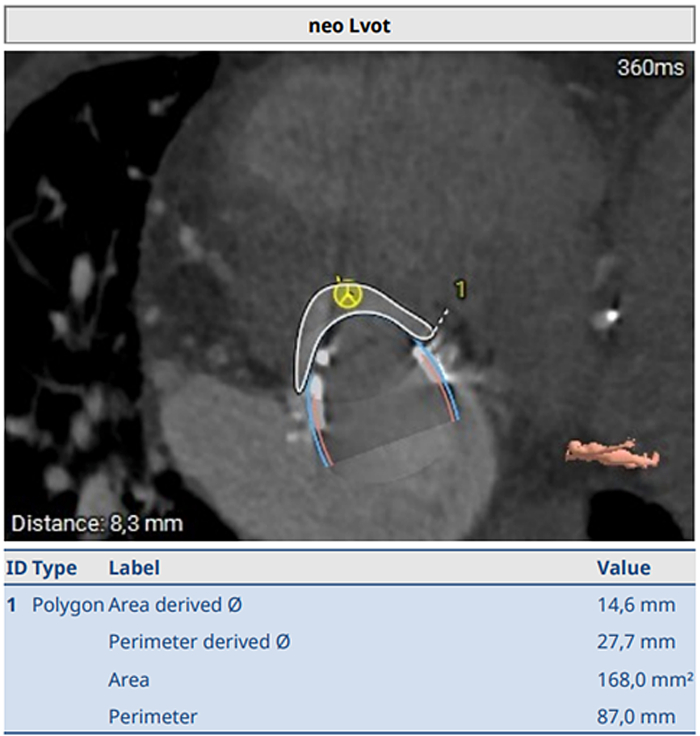
Computed Tomography Assessment of Neo-LVOT Before Mavacamten Computed tomography–based measurement of the predicted neo-LVOT area before mavacamten therapy, measuring 168 mm^2^. LVOT = left ventricular outflow tract.

On index admission, transthoracic and transesophageal echocardiography confirmed severe restenosis of the surgical bioprosthesis (mitral valve area: 0.93 cm^2^, mean transmitral gradient: 14 mm Hg) (Figures 1 and 2) with preserved left-ventricular function (62%) and size (left ventricular end-diastolic diameter: 44 mm, left ventricular end-systolic diameter: 34 mm, interventricular septal thickness: 13 mm). Coronary angiography demonstrated stable coronary disease with patent bypass grafts. A dedicated screening cardiac CT for TMViV predicted a neo-LVOT area of 168.0 mm^2^ (Figure 3). At that time, echocardiographic Doppler interrogation did not show a relevant LVOT gradient (maximum pressure gradient: 10 mm Hg), but CT-based prediction indicated prohibitive risk for postprocedural LVOTO, consistent with published evidence linking a small predicted neo-LVOT to LVOTO and adverse outcomes after TMVR.^2^, ^3^, ^4^, ^5^

Given the high risk of LVOTO and the patient's prohibitive surgical risk, the heart team reviewed established LVOTO-mitigation strategies, including anterior leaflet modification (LAMPOON) and pre-emptive septal reduction with alcohol septal ablation (ASA). However, neither approach was considered appropriate or beneficial in this patient within our multidisciplinary risk-benefit assessment. Therefore, the heart team opted for an off-label, pharmacologic strategy aimed at reducing risk of LVOTO. Mavacamten 2.5 mg once daily was initiated. Therapy was accompanied by structured echocardiographic surveillance, in line with established monitoring principles for myosin inhibition.

**Figure 4:**
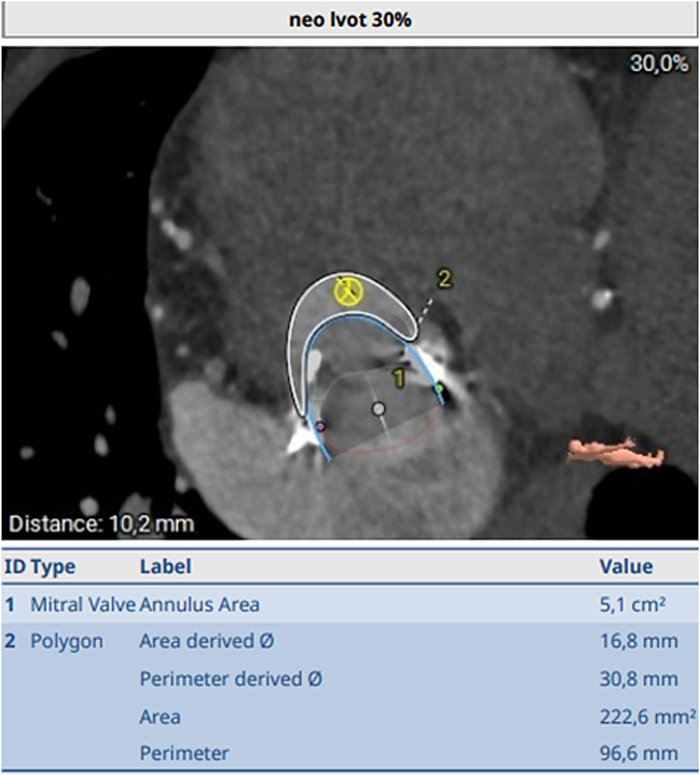
Computed Tomography Assessment of Neo-LVOT During Mavacamten Therapy Computed tomography–based measurement of the predicted neo-LVOT area during mavacamten therapy, measuring 222.6 mm^2^. LVOT = left ventricular outflow tract.

Repeat screening CT (after 4 months of mavacamten) indicated a reduced risk of LVOTO, with an estimated neo-LVOT area of 222.6 mm^2^ (Figure 4), exceeding our cutoff of >200 mm^2^ and moving beyond commonly reported high-risk thresholds (∼170-190 mm^2^) used in TMVR screening frameworks.

**Figure 5:**
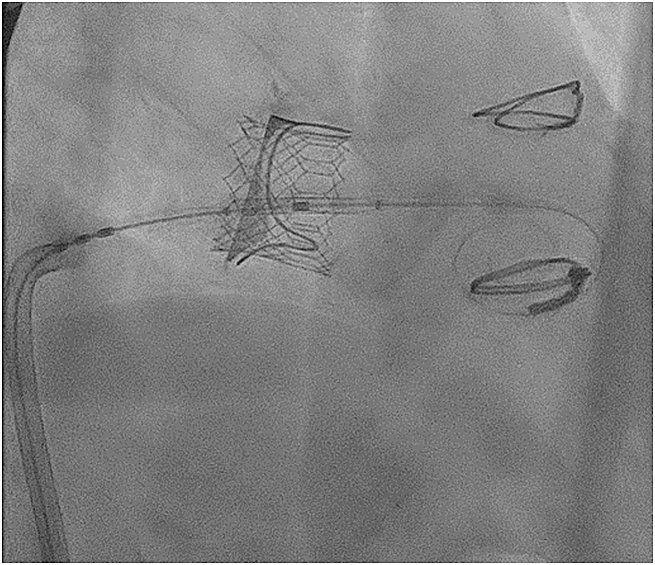
Fluoroscopic View After Implantation of the Balloon-Expandable Transcatheter Heart Valve Fluoroscopic view after deployment of the balloon-expandable valve in the expected position within the degenerated surgical bioprosthetic mitral valve.

**Figure 6:**
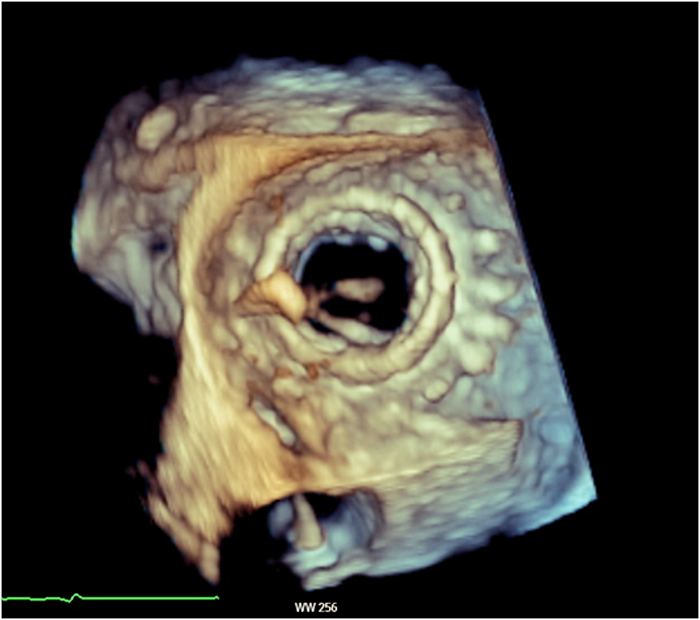
3-Dimensional Transesophageal Echocardiography of the Mitral Valve Prosthesis After Deployment Three-dimensional transesophageal echocardiography of the mitral valve after deployment of the balloon-expandable valve demonstrates an excellent result and no paravalvular leak.

**Figure 7:**
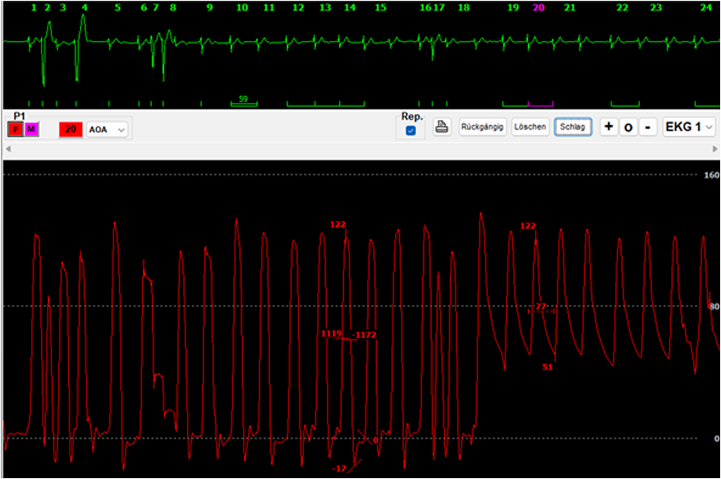
Invasive Hemodynamics After Deployment Invasive hemodynamics and pressure measurements from the left ventricle to the aorta after deployment indicating no relevant pressure gradient.

The patient was admitted electively for planned TMViV. A transseptal TMViV was performed under analgosedation without general anesthesia. A 26-mm Sapien 3 transcatheter valve (Edwards Lifesciences) was implanted successfully within the degenerated surgical bioprosthesis (Figure 5). Total procedural time was approximately 60 minutes. No procedural complications occurred. Intraprocedural transesophageal echocardiography showed an excellent immediate result with no paravalvular leak and no evidence of clinically relevant LVOTO (Figure 6). Invasive hemodynamics demonstrated no relevant pressure gradient between the left ventricle and aorta (Figure 7).

**Figure 8:**
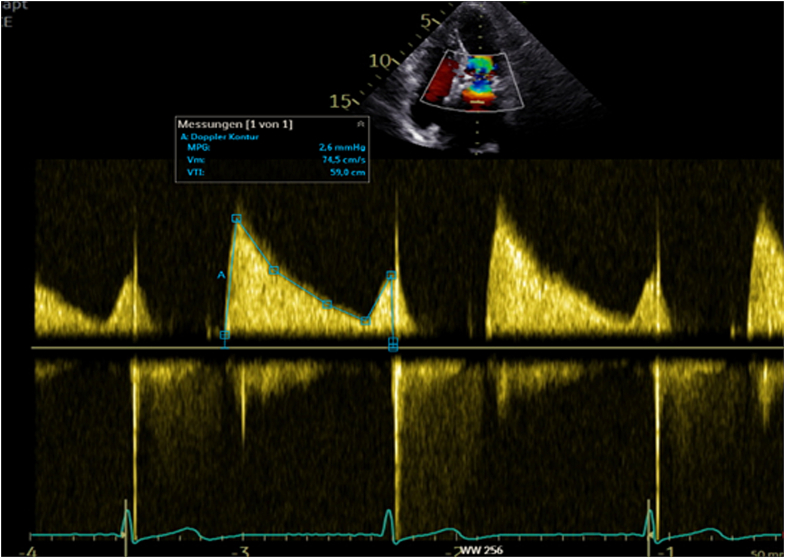
Transthoracic Echocardiography After Intervention Transthoracic echocardiography of the mitral valve after valve-in-valve procedure; continuous-wave Doppler across the mitral valve shows a mean transmitral gradient of 2.6 mm Hg.

Postprocedural transthoracic echocardiography confirmed normal TMViV function without regurgitation or paravalvular leak, mean transmitral gradient of 2.6 mm Hg, and preserved left ventricular function (left ventricular ejection fraction [LVEF]: 57%) without relevant LVOTO (Figure 8). The patient was monitored in the intermediate care unit for 1 day, then transferred to a standard ward and discharged home after 4 days.

## Why Beyond the Guidelines

Current TMVR pathways rely on CT-predicted neo-LVOT to identify prohibitive LVOTO risk and mainly propose procedural mitigation strategies rather than pharmacologic preconditioning. In this patient, redo surgery and established LVOTO-mitigation procedures were not feasible, so off-label mavacamten with structured echocardiographic surveillance was used to increase the predicted neo-LVOT and enable transseptal valve-in-valve TMVR.

## Outcome and Follow-Up

The patient was discharged after the intervention without continued mavacamten therapy. At the 30-day follow-up, she showed a favorable clinical course with markedly improved symptoms. Echocardiography continued to show no evidence of LVOTO.

## Discussion

This case highlights a pragmatic clinical problem in contemporary structural heart interventions: A failed surgical bioprosthetic mitral valve in elderly, high-risk patients where TMViV is otherwise attractive but CT screening predicts high risk of LVOTO.

The concept of the neo-LVOT—the residual LVOT corridor after TMVR created by the transcatheter valve frame and displaced leaflet tissue—is foundational for risk prediction.^2^ Expert recommendations emphasize standardized CT segmentation and virtual valve implantation to quantify neo-LVOT.^3^^,^^4^ A practical limitation is that the predicted neo-LVOT is not a fixed truth but a model-derived measurement; particularly near decision thresholds, modest differences in segmentation, cardiac phase selection, or assumed valve position during virtual implantation can shift a patient across high-risk cutoffs. This reinforces the need to reduce measurement uncertainty through standardized workflows and, in borderline cases, repeat planimetry or an independent second read to support heart team decision-making. Observational data show that a small predicted neo-LVOT strongly predicts LVOTO and is associated with markedly higher procedural mortality.^5^ Reported cutoffs vary by methodology and cohort; thresholds around <170 to 189 mm^2^ have been linked to high sensitivity for LVOTO.^5^^,^^11^

In our patient, the predicted neo-LVOT area of 168 mm^2^ was in a high-risk range despite the absence of a measured resting LVOT gradient on Doppler. This discrepancy is clinically plausible because LVOTO after TMVR can be dynamic and is driven by altered geometry after implantation rather than baseline hemodynamics.

Several interventional strategies can mitigate LVOTO risk. These include anterior leaflet modification, most notably LAMPOON, which intentionally splits the anterior mitral leaflet to a percentage of the LVOT encroachment and has shown acceptable outcomes in select candidates.^12^ It is most compelling when LVOTO is driven by anterior leaflet displacement, but it also requires favorable anatomy and procedural expertise. Septal reduction—most commonly, pre-emptive ASA performed weeks before TMVR to allow septal remodeling—can increase predicted neo-LVOT and enable TMVR in patients initially screened out owing to prohibitive LVOTO risk.^13^ ASA requires favorable coronary anatomy, specifically an appropriate septal perforator supplying the target basal septum, and it is therefore not feasible in all patients (eg, absent/small or unfavorably distributed septal branches).^13^^,^^14^ These constraints may leave a subset of patients without a practical procedural LVOTO-mitigation option despite otherwise favorable TMViV candidacy.

Because no alternative LVOT mitigation strategy was considered feasible, our heart team opted for a noninvasive approach with mavacamten to reduce septal contribution/hypercontractility and improve the predicted outflow tract corridor. Compared with focal anatomical strategies (LAMPOON, ASA), myosin inhibition targets global myocardial contractility and may offer a noninvasive “bridge” when procedural mitigation is not feasible.

Mavacamten has proven efficacy in obstructive HCM, improving symptoms and reducing LVOT gradients, with evidence of favorable structural effects on imaging.^8^, ^9^, ^10^ Importantly, its use mandates careful LVEF monitoring given the risk of systolic dysfunction and may be limited in patients with impaired baseline left ventricular function or in those who develop a decline in LVEF during therapy, and prespecified thresholds for interruption.^6^^,^^7^

Although our patient did not have the classic obstructive HCM phenotype, the mechanism of action—reducing excessive myosin-actin cross-bridge formation and contractility—can, in principle, influence left ventricular geometry and systolic septal contribution. Trial data and imaging substudies confirm that remodeling effects are not purely hemodynamic and can be accompanied by reductions in left ventricular mass and improvements in filling indices over time.^8^, ^9^, ^10^

After 4 months of therapy, the predicted neo-LVOT in our case increased to 222.6 mm^2^, enabling TMViV with excellent acute results without LVOTO. However, outside a classic obstructive HCM phenotype, the optimal treatment duration and preprocedural monitoring schedule for this off-label strategy remain uncertain. We therefore aligned follow-up with established obstructive HCM pathways: clinical review and echocardiography assessing LVEF at baseline, weeks 4, 8, and 12, and then every 12 weeks, as pharmacogenetic evaluation may help estimate mavacamten metabolism. Given the unknown dosing strategy in this setting, we used a conservative low-dose regimen (2.5 mg once daily), and future studies should define how duration and dosage relate to baseline obstruction risk and the neo-LVOT gain required.

## Conclusions

This case shows that CT-predicted LVOTO risk can preclude transseptal TMViV despite absent baseline Doppler obstruction. In a highly selected high-risk patient, off-label low-dose mavacamten with echocardiographic surveillance increased predicted neo-LVOT and enabled uncomplicated TMViV without postimplant LVOTO. Mavacamten may serve as a noninvasive “bridge to TMVR” when established LVOTO-mitigation strategies are not feasible; prospective studies should define selection, monitoring, and durability.

## Funding Support and Author Disclosures

Dr Polzin has declared lecture honoraria and advisory board activities for Abbott and Edwards Lifesciences. All other authors have reported that they have no relationships relevant to the contents of this paper to disclose.

**Take-Home Messages**

• Myosin inhibition with mavacamten may increase CT-predicted neo-LVOT and enable TMVR when surgery and procedural LVOT obstruction mitigation are not feasible.
• Careful patient selection and close imaging surveillance are essential for safety and durability.
